# Traditional Chinese Medicine Constitution Identification Based on Objective Facial and Tongue Features: A Delphi Study and a Diagnostic Nomogram for Blood Stasis Constitution

**DOI:** 10.1155/2022/6950529

**Published:** 2022-03-29

**Authors:** Yue Meng, Li'an Liu, Yanfei Zheng, Lingru Li, Xing Liu, Junjun Qin, Jing Xia, Diankun Cui, Jinfeng Liang, Zhuqing Li, Tianxing Li, Taotao Wu, Yun Yan, Wenle Li, Yaoyao Zhou, Jianxiang Sun, Shujuan Hou, Qi Wang

**Affiliations:** ^1^National Institute of Traditional Chinese Medicine Constitution and Preventive Treatment of Diseases, Beijing University of Chinese Medicine, Beijing 100029, China; ^2^College of Chinese Medicine, Beijing University of Chinese Medicine, Beijing 100029, China; ^3^College of Chinese Classics, Beijing University of Chinese Medicine, Beijing 100029, China; ^4^Institute of Basic Theory for Chinese Medicine, China Academy of Chinese Medicine Science, Beijing 100700, China; ^5^School of Life Sciences, Beijing University of Chinese Medicine, Beijing 100029, China

## Abstract

**Objective:**

The aim of this study was to systematically summarize and form an expert consensus on the theoretical experience of tongue and facial features for the identification of nine types of traditional Chinese medicine (TCM) constitution. Additionally, we sought to explore the feasibility of TCM constitution identification through objective tongue and facial features.

**Methods:**

We used Delphi method to investigate the opinions of experts on facial and tongue feature items for identifying TCM constitution. We developed and validated a diagnostic nomogram for blood stasis constitution (BSC) based on objective facial and tongue features to demonstrate the reliability of expert consultation.

**Results:**

Eleven experts participated in two rounds of expert consultation. The recovery rates of the two rounds of expert consultation were 100.0% and 90.9%. After the first round, 39 items were screened out from 147 initial items, and 2 items were supplemented by experts. In the second round, 7 items were eliminated, leaving 34 items for 8 types of TCM constitution. The coefficient of variation in the first round was 0.11–0.49 for the 147 items and 0.11–0.29 for the included items. The coefficient of variation in the second round was 0.10–0.27 for the 41 items and 0.10–0.20 for the included items. The *W* value was 0.548 (*P* < 0.001) in the first round and 0.240 (*P* < 0.001) in the second round. Based on expert consultation, we selected BSC as an example and developed and validated a diagnostic nomogram consisting of six indicators: sex, hair volume, lip color-dark purple, susceptibility-facial pigmentation/chloasma/ecchymosis, zygomatic texture-red blood streaks, and sublingual vein-varicose and dark purple. The nomogram showed good discrimination (AUC: 0.917 [95% confidence interval [CI], 0.877–0.956] for the primary dataset, 0.902 [95% CI, 0.828–0.976] for the validation dataset) and good calibration. Decision curve analysis demonstrated that the nomogram was clinically useful.

**Conclusion:**

This is the first study to systematically summarize the existing knowledge and clinical experience to form an expert consensus on the tongue and facial features of nine types of TCM constitution. Our results will provide important prior knowledge and expert experience for future constitution identification research. Based on expert consultation, this study presents a nomogram for BSC that incorporates objective facial and tongue features, which can be conveniently used to facilitate the individualized identification of BSC.

## 1. Introduction

Traditional Chinese medicine (TCM) has been widely used in health maintenance and disease treatment for thousands of years. TCM emphasizes treatment chosen according to the variability of an individual, known as personalized treatment. Based on this conception, TCM constitution was advanced to describe the variability of the holistic body condition. In April 2009, the China Association of Chinese Medicine (CACM) published a professional standard to delimit the classification of the nine following TCM constitution types: balanced constitution (BC), Qi-deficiency constitution (QDC), Yang-deficiency constitution (YADC), Yin-deficiency constitution (YIDC), phlegm-dampness constitution (PDC), damp-heat constitution (DHC), blood stasis constitution (BSC), Qi-stagnation constitution (QSC), and inherited-special constitution (ISC) [1]. Individuals with different constitution types have different disease susceptibilities and tendencies [2–4]. Constitution classification can provide a prediction of the future development of certain diseases, and intervention for a pathological constitution can act accordingly as a way of prevention. Therefore, identifying individuals' TCM constitution can promote effective health management and significantly benefit the application of personalized medicine [5, 6].

At present, the Constitution in Chinese Medicine Questionnaire (CCMQ), which contains items related to subjective symptoms and judgment, is the main tool of constitution identification [7, 8]. The CCMQ has good reliability and validity [9] and is widely used in scientific research and clinical practice. However, it has some shortcomings [10, 11]. Firstly, it is a self-assessment scale and there are differences in individual understanding of items and scores. Moreover, the lack of professional backgrounds of patients often has an unavoidable influence on the judgment of the constitution result. Therefore, the objective qualitative and quantitative analysis of the relatively stable external appearance of the human body can greatly reduce the deviation of the results of constitution identification caused by subjective factors. Secondly, the CCMQ comprises 60 questions, and it is time-consuming to complete the questionnaire. As TCM constitution has entered the national public health service system as the only physical examination item in TCM, the identification technology of constitution faces a wider range of clinical, preventive, and healthcare needs. As a result, the constitution identification technology needs to be upgraded to be more convenient and objective.

With the continuous advancement of detection methods and intelligence technology, the research on the methods of constitution identification is constantly advancing toward objectification. Not only are tongue and facial features important in the inspection of TCM but also they play an important role in identifying TCM constitution. There are 17 questions related to external signs in the CCMQ, accounting for 28.3% of the total questions. Among these questions, there are 11 questions about facial features, accounting for 64.7% of the total number of questions about external signs, for example, “were your lips redder than others?,” “did you get hot flashes?,” and “did you have a darkened facial skin or get brown spots easily?” [12]. Meanwhile, tongue and face images are easy to collect, and facial recognition technology is maturing. Recognition by tongue image and facial features can be used as an auxiliary means of the CCMQ, reducing the number of questions and the time for answering questions and laying the foundation for developing multidimensional indicators of TCM constitution identification tools.

Therefore, tongue and facial features have become a hotspot in the intelligent identification of TCM constitution. In 2017, Huan et al. [13] proposed an algorithm based on a deep convolutional neural network, which could classify individual constitution types according to face images with an accuracy of 65.29%. In 2019, Huan and Wen [14] proposed a multilevel and multiscale feature aggregation method within the convolutional neural network with an accuracy reaching 69.61%. Furthermore, Zhang et al. [15] developed a dynamic classification model of facial skin and TCM constitution, which could effectively identify the complex relationship between the facial skin index and the internal type of TCM constitution by utilizing data information involved in modeling. Ma et al. [16] presented a system framework to automatically identify the constitution through natural tongue images, where deep convolutional neural networks were carefully designed for tongue coating detection, tongue coating calibration, and constitution recognition. Pan et al. [17] extracted and analyzed the characteristics of the objective tongue images (the features of hue and saturation of the tongue body and tongue coating, average intensity, smoothness of tongue coating texture, and tongue marks) and body features to develop an auxiliary TCM constitution identification model of an artificial neural network and support vector machine. Throughout the research in this field, two weak points have been found: (1) Lack the guidance of the prior knowledge of facial features of TCM constitution, directly use a certain number of tongue and face images with constitution labels for machine learning, and pursue the maximization of accuracy through different modeling methods. In the absence of a large sample size and a sufficient number of experts who conduct accurate constitution labeling, this end-to-end feature learning framework will likely lead to poor extrapolation and be unable to withstand real-life verification. (2) some studies [18–21] have drawn on the knowledge and expert experience of the tongue or facial features of TCM constitution. However, due to a large number of inconsistencies and a lack of uniform standards, this knowledge cannot play an accurate and reliable guiding role during the process of constitution identification modeling. The aforementioned shortcomings reveal the significance of standardization and consensus on the expert experience of tongue and facial features in TCM constitution identification.

Here, we used the Delphi method to conduct an expert consultation on facial and tongue feature items for TCM constitution identification. Then, we chose the BSC as an example to demonstrate that facial features have the greatest contribution to identification, which was used to build a diagnostic model based on the expert scoring of facial and tongue features obtained from the Delphi conclusion. Facial images were also collected by a self-developed machine with a fixed light source to review the facial features of the participants, to display typical facial images, and to accumulate raw data for future research on constitution identification through image recognition.

## 2. Methods

This study included two phases: Phase 1, which used the Delphi method to investigate the opinions of experts on facial feature items for identifying TCM constitution and, through the integration of opinions, established the facial feature items with diagnostic value, and Phase 2, which focused on the constitution type, of which the facial features have a high contribution to the diagnosis. We recruited participants of this constitution type and developed and validated a diagnostic nomogram based on objective facial and tongue features.

### 2.1. Development of the Item Pool of Facial and Tongue Features in TCM Constitution Identification

Literature research was conducted on the tongue and facial features of each constitution type, and items from textbooks, monographs, standards, journals, dissertations, and other literature were extracted. The data retrieval time was March 8, 2021. The searched databases included China National Knowledge Infrastructure (CNKI) Database, Wanfang Database, China Science and Technology Journal Database (CQVIP), and PubMed. Book retrieval was performed using Superstar Huiya e-book database (https://www.sslibrary.com). The search terms included “constitution,” “physique,” “Chinese medicine,” “tongue,” “face,” “complexion,” “color,” and “facial shape.” The Chinese Academy of Chinese Medical Science Clinical Terminology System (https://tcmcts.org/tpweb/CommonAction_main, CTS) was also searched. Regarding the searched books and journals, via the preliminary screening of the title and abstract, the full texts were browsed one by one, and information on facial and tongue features was extracted from the papers that met the nine constitution classification types to an Excel table to form the item pool. The initial questionnaire for expert consultation was consistent with the item pool.

### 2.2. Adjusting Facial and Tongue Feature Items Using the Delphi Method

The Delphi survey technique is a consensus method to help enhance effective decision-making in health and social care [22] and is primarily used when the available knowledge is incomplete or subject to uncertainty [23]. After the item pool was developed, the Delphi method was adopted to evaluate and select the items with good diagnostic value.

#### 2.2.1. Expert Consultation Panel

The expert inclusion criteria were as follows: (1) engaging in TCM-related work for more than 5 years, with rich theoretical knowledge and clinical experience; (2) majoring in TCM constitution, TCM prevention and treatment of disease, TCM diagnostics, or related profession; (3) having relevant research or clinical experience in TCM constitution identification; and (4) willing to participate in the expert consultation.

#### 2.2.2. Expert Consultation Process

The invitation letter and the first round of expert consultation questionnaires were sent by e-mail to experts who met the inclusion criteria, with the number of experts controlled at 10–15.

The questionnaire of the first round included an introduction to the research background, instructions for completing the questionnaire, general information pertaining to the expert, and a scoring column for the value of importance of each item. The 5-point Likert rating method was used to determine the importance degree of item, which was set as “critical,” “important,” “neutral,” “unimportant,” and “very unimportant” and was rated 5–1 points, respectively. Additionally, the first round of the questionnaire posed 10 questions regarding the overall evaluation: “What do you think the importance of facial and tongue features is in the identification of TCM constitution and in each of the nine constitution types?” The experts were asked to select scores from 0 points as “very unimportant” to 10 points as “critical.”

All of the outcomes of the first round were summarized and analyzed using the following item inclusion criteria: (1) mean score of importance value of the item ≥4.0 points or the proportion of experts who rated the item ≥4.0 points was >75%; (2) item had no objection from the research group. Items of each round were adjusted based on the inclusion criteria and the selected items formed the next round of the expert consultation.

In the second round of the expert questionnaire, in addition to the importance value, experts were asked to evaluate the familiarity degree of each item. Familiarity degree was set as “very familiar,” “familiar,” “neutral,” “unfamiliar,” and “very unfamiliar” and was rated 1.0 point, 0.8 points, 0.5 points, 0.2 points, and 0.0 points, respectively. The expert was expected to judge items based on theoretical analysis, practical experience, domestic and foreign references, and subjective intuition, each of which can be divided into “large,” “medium,” and “small” according to the influence degree of judgment. The sum of the scores from these four parts formed the judgment coefficient (Table 1) [24].

Both rounds of the questionnaire set up questions to ask the expert if they have additional advice. When the opinions of all experts tended to be the same, the facial and tongue feature items for constitution identification were confirmed. In most studies, two rounds of expert consultation were used.

#### 2.2.3. Statistical Analysis

The data were collected by Wenjuanxing web application via e-mail. SPSS version 20 and Excel 2019 were applied to further analyze the data. Data are presented as the mean ± standard deviation or *n* (%).

### 2.3. Development of a Diagnostic Nomogram of BSC Based on Facial and Tongue Features Based on Expert Consultation Results

After expert consultation in the previous phase, we selected BSC as an example and developed and validated a diagnostic nomogram based on facial and tongue feature items.

#### 2.3.1. Participants

A cross-sectional study was conducted from June to August 2021 in Beijing, China. A total of 296 participants were recruited by a professional consulting company. The inclusion criteria were as follows: 18–64 years old, balanced constitution or BSC, and signing informed consent. The exclusion criteria were as follows: more than one type of constitution; history of facial surgery, prosthesis implantation, or trauma; patients with severe audiovisual, language impairment, or severe mental illness, which affects the correct understanding and answers to the questionnaire; experience of major illness in the past year; pregnancy or childbirth, which may cause major changes in TCM constitution; and being in the acute phase of the disease, which may cause significant changes in complexion. The participants were randomly divided into a primary group and a validation group at a ratio of 7 : 3.

#### 2.3.2. Information Collection Progress of TCM Constitution, Facial and Tongue Features, and Images

  Step 1: Recruitment advertisements were issued by consulting companies on the Internet. Participants were asked to fill in general information, including age, sex, height, and weight, and complete the CCMQ to identify the TCM constitution. If the result was BC or BSC and the participant met the inclusion criteria, the preliminary screening was passed.
  Step 2: Participants came to the clinical trial site at their appointment time, and an experienced TCM expert conducted constitution identification using the four diagnostic techniques. If the constitution result met BC or BSC, there were no other constitution types mixed, and the exclusion criteria were not met, the participant was confirmed to be included in the BC group or the BSC group.
  Step 3: According to the Delphi study result, the feature items of BSC form an expert scoring table, where the score of each item ranges from 0 to 10, and the visual analog scale is applied: 0 points meant that there was no such feature (0%), while 10 points represented that this feature was very typical (100%). To reduce the bias caused by the doctor's subjectivity, we set up three TCM experts to simultaneously score the facial and tongue features of a participant without knowing the constitution type. We removed the lowest and highest score and chose the middle score as the scoring result of the corresponding facial feature. Before scoring, participants were asked to remove their makeup and ensure that they did not engage in strenuous exercise in the prior 30 min.
  Step 4: Face and tongue images were collected by a self-developed image collection machine. Because the color of the tongue and face is a critical piece of diagnostic information of Chinese medicine, this machine was designed to have a fixed light source. When the face is placed on the machine, a closed environment is formed to avoid interference from external light. Images of face, upper tongue, and sublingual veins were collected.

#### 2.3.3. Statistical Analysis

Statistical analysis was conducted with R software version 3.6.0 and STATA version 15.0. The reported statistical significance levels are all two-sided, with statistical significance set at 0.05.

*(1) Demographic Comparison between BSC and Non-BSC in the Primary and Validation Groups*. Wilcoxon rank-sum test was used to assess the differences in age, while Chi-squared (*χ*^2^) tests were used to compare the difference in categorical variables (sex, BMI level, hair features, and facial/tongue features) between the participants with BSC and BC in both the primary and validation datasets.

*(2) Feature Selection and Diagnostic Nomogram Building*. The least absolute shrinkage and selection operator (LASSO) method, which is suitable for the regression of high-dimensional data [25], was used to select the most useful diagnostic features from the primary dataset. We used a multivariate binary logistic regression model of BSC consisting of features selected by LASSO. To provide the clinician with a quantitative tool to diagnose individual probability of BSC, the multivariable logistic model was presented as a nomogram.

*(3) Validation of the Nomogram in the Primary and Validation Datasets*. The developed nomogram was validated with respect to the discrimination ability using area under the receiver operating characteristic (ROC) curve (AUC), the calibration ability using the calibration curve accompanied by Hosmer-Lemeshow (H-L) chi-square statistics (a significant test statistic implies that the model does not calibrate perfectly [26]), and the clinical use, using decision curve analysis (DCA). Discrimination refers to the ability of a model to correctly distinguish nonevents and events. Calibration measures how closely the predicted probabilities agree numerically with the actual outcomes [27]. DCA was conducted to determine the clinical usefulness of the diagnostic nomogram by quantifying the net benefits at different threshold probabilities [28]. The three dimensions of evaluating the nomogram were conducted in both the primary and validation datasets.

#### 2.3.4. Ethical Approval and Clinical Trial Registry

The study was approved by the Institutional Review Board of the Ethics Committee of Beijing University of Chinese Medicine (2021BZYLL0107), in accordance with the principles in the Declaration of Helsinki. All participants provided written informed consent. The trial is registered with the Chinese Clinical Trial Registry (ChiCTR2100048289) (https://www.chictr.org.cn/).

## 3. Results

### 3.1. Expert Consultation

#### 3.1.1. Initial Consultation Questionnaire

Through a literature study, we screened out one professional standard, one monograph, 11 journal papers, three dissertations, and one terminology system. After the process of extracting tongue and facial feature items, eight regional locations of tongue and facial features were initially established: the whole face, forehead, zygomatic, cheek, eye, nose, lips, and tongue. A total of 147 entries belonging to nine constitution types were extracted into the item pool; there is a possibility of conflict between these entries. Due to the large number of items, the initial questionnaire of the first round was designed to ask experts to evaluate the importance value to screen out a batch of important items, without familiarity degree evaluation.

#### 3.1.2. Two Round of Expert Consultation

In this study, two rounds of expert consultation were adopted, enrolling 11 experts. The detailed expert information is listed in Table 2. The recovery rates of the expert consultation questionnaire were 100% in the first round and 90.9% in second round.

In the first round, 11 experts were asked to make an overall evaluation of the importance of facial and tongue features in the identification of TCM constitution and nine types of constitution.  Table 3 shows that the mean importance value of facial and tongue features to TCM constitution identification was 7.73 points, in which BSC had the highest score (8.91 points). We believe that this score indicates that the facial and tongue features are important for constitution identification and contribute differently to different constitution types.

The degree of concentration of expert opinions was analyzed by the importance value and coefficient of variation. The greater the importance assignment, the smaller the coefficient of variation value, indicating that the degree of expert opinion concentration was better [29]. The degree of concentration of expert opinions is shown in Table 4. After the first round of importance evaluation, 39 items were screened out, and the experts provided two supplementary items, which were added to the questionnaire. In the second round, experts scored the importance value, familiarity degree, and judgment basis of 41 items. Finally, 7 items were eliminated in the second round, with 34 items remaining: 11 items for BSC, 6 for DHC, 5 for PDC, 4 for BC, 4 for YIDC, 2 for YADC, 1 for QSC, and 1 for ISC. No items for QDC remained following screening.

The coefficient of variation in the first round was 0.11–0.49 in all items and 0.11–0.29 in included items, and, in the second round, it was 0.10–0.27 in all items and 0.10–0.20 in included items. As the coefficients of variation of the items selected from the second round were all <0.25, it was considered that the concentration of the experts was good, and there was no need to conduct a third round of consultation. The coefficient authority of expert was determined as (expert judgment coefficient + expert familiarity degree)/2. The coefficient of authority in the second round of expert consultation was 0.77–0.97, and the mean value was 0.9. Kendall's *W* was adopted to judge the degree of expert opinion coordination. The value of Kendall's *W* was between 0 and 1, with a higher value indicating that the opinion of the expert was more consistent and the coordination of the questionnaire was better [29]. The *W* value was 0.548 (significance test *χ*^2^ = 885.688, *P* < 0.001) in the first round and 0.240 (significance test *χ*^2^ = 96.860, *P* < 0.001) in the second round. The large difference in *W* values between the two rounds was likely because the number of items and the number of experts varied between rounds.

Through the Delphi study, we have obtained an expert consensus. First, the importance of facial and tongue features for identification of each TCM constitution is different. Second, BSC, PDC, and DHC have relatively more representative facial and tongue features. Most importantly, we generated a facial and tongue feature item table containing 34 items for 8 types of TCM constitution, which can provide an expert consensus reference for related research.

### 3.2. Development and Validation of the BSC Nomogram Based on Facial and Tongue Features

To further verify the results of expert consultation, we chose BSC as an example to build a diagnostic model through objective facial and tongue features and presented this as a nomogram to provide a tool for clinical use.

#### 3.2.1. Participants' Characteristics

The characteristics of the participants in the primary and validation groups are given in Table 5. A total of 296 participants were included and were randomly divided into the primary group (*n* = 214) and validation group (*n* = 82), with 28.97% and 23.17% BSC prevalence, respectively. The primary group showed that sex (*P* < 0.01) and age (*P* < 0.01) differed between the BSC and non-BSC groups. Women and the elderly were more inclined to be in BSC status. To enrich the objective appearance diagnostic information, we added BMI and hair features, which were obtained from participants who completed the information on height, weight, hair volume, and hair oil secretion. The percentage of less hair volume (*P* < 0.01) and dry hair (*P* < 0.01) was higher in the BSC group than in the non-BSC group, while the BMI was not significantly different between groups. In terms of the facial and tongue features, we made a slight adjustment to the 11 BSC items obtained from the previous expert consultation; that is, facial pigmentation, chloasma, and ecchymosis were combined into one item, because these features are similar and not easy to distinguish. Therefore, 9 indicators of face and tongue features were formed and scored by three experienced TCM practitioners. In the primary group, all facial and tongue features showed significant differences between the BSC and non-BSC groups.

#### 3.2.2. Feature Selection and Diagnostic Nomogram Construction

**Figure 1:**
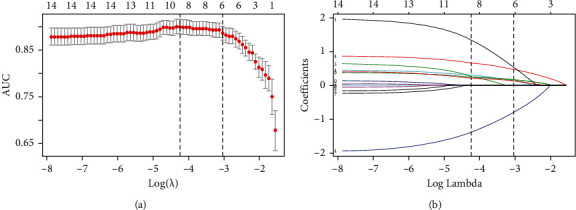
Predictor selection using the least absolute shrinkage and selection operator (LASSO) logistic regression model. (a) Tuning parameter (*λ*) selection in the LASSO model used 10-fold cross-validation. The area under the receiver operating characteristic (AUC) curve was plotted versus log (*λ*). Dotted vertical lines were drawn at the optimal values by using the maximum criteria and the 1 standard error of the maximum criteria (the 1-SE criteria). (b) LASSO coefficient profiles of the 14 feature indicators. The dotted vertical line was plotted at the value selected using 10-fold cross-validation in figure (a). For the 1-SE criteria, the optimal *λ* resulted in six nonzero coefficients.

**Figure 2:**
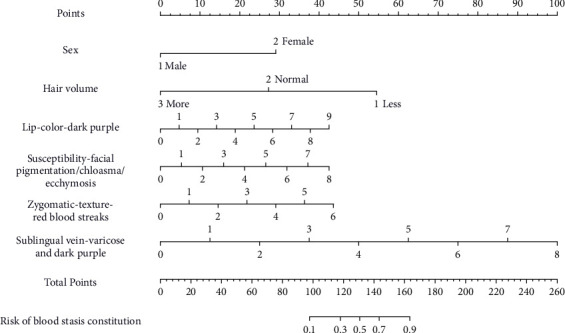
Developed diagnostic nomogram for blood stasis constitution. The nomogram was developed in the primary group, with six indicators incorporated.

The logistic LASSO model is a shrinkage method that can actively select from a large and potentially multicollinear set of variables in the regression, resulting in a more relevant and interpretable set of predictors [30]. The most prominent advantage of LASSO regression is that, by penalized regression on all variable coefficients, the coefficients of relatively unimportant independent variables become 0 and are therefore excluded from modeling. We used 10-fold cross-validation to select the penalty term, *λ*. Two *λ* were produced—one that maximizes the AUC and one representing the largest *λ* that is still within 1 standard error of the maximum AUC. We opted for the latter *λ* as it results in stricter penalty allowing us to reduce the number of covariates even further than the former *λ* [31]. After variable selection by LASSO regression, sex, hair volume, lip-color-dark purple, susceptibility-facial pigmentation/chloasma/ecchymosis, zygomatic-texture-red blood streaks, and sublingual vein-varicose and dark purple were selected as the best subset of feature indicators to develop the BSC diagnostic model (Figures 1(a) and 1(b)). The multivariate binary logistic regression model for identifying BSC was developed taking the selected indicators into account. The model was visualized as a nomogram (Figure 2). The nomogram is used by summing all points identified on the scale for each variable, where the total points projected on the bottom scales indicate the risk of BSC.

#### 3.2.3. Validation of the Nomogram in the Primary and Validation Groups

**Figure 3:**
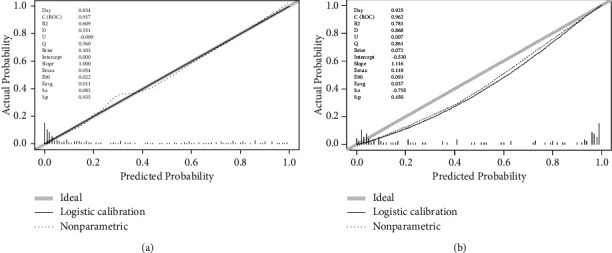
(a) Calibration curve for primary data. (b) Calibration curve for validation data.

**Figure 4:**
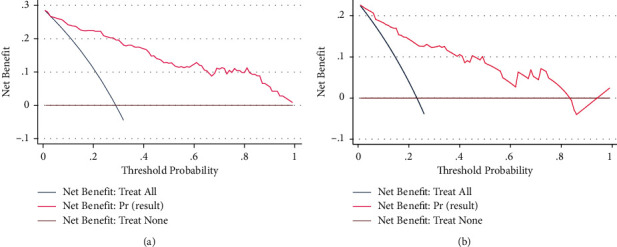
(a) Decision curve for primary data. (b) Decision curve for validation data. The vertical axis represents the value of net benefit, and the horizontal axis represents the threshold level. Plotting net benefit in function of threshold level yields the decision curve. The blue line is the net benefit of treating all, the purple line is the net benefit of treating none, and the pink line is the net benefit of treating based on the BSC nomogram. The abscissa of the two intersection points (pink and blue, pink and purple) represents within the threshold range, the application of this nomogram is clinically useful.

The AUC of the diagnostic nomogram was 0.917 (95% CI, 0.877–0.956) for the primary dataset and 0.902 [95% CI, 0.828–0.976] for the validation dataset, which indicated good discrimination and good robustness for the nomogram. The calibration curves for the primary and validation datasets are shown in Figures 3(a) and 3(b). Calibration curves depict the calibration of each model in terms of the agreement between the diagnosed risks of BSC and actual outcomes of BSC. The calibration curve was evaluated by the unreliability *U* test. The nomogram was well calibrated with no significant difference between the predicted probability and the actual probability (*P* = 0.935 in primary data and *P* = 0.450 in validation data). The Hosmer-Lemeshow test yielded a nonsignificant statistic (*P* = 0.295 when dividing primary data into default 10 groups, *P* > 0.159 when dividing validation data into <9 groups) which indicated that the degree of calibration is acceptable. The DCA for the BSC diagnostic nomogram is presented in Figures 4(a) and 4(b), which indicated good clinical applicability of the nomogram.

#### 3.2.4. Image Display of Typical Facial and Tongue Features

**Figure 5:**
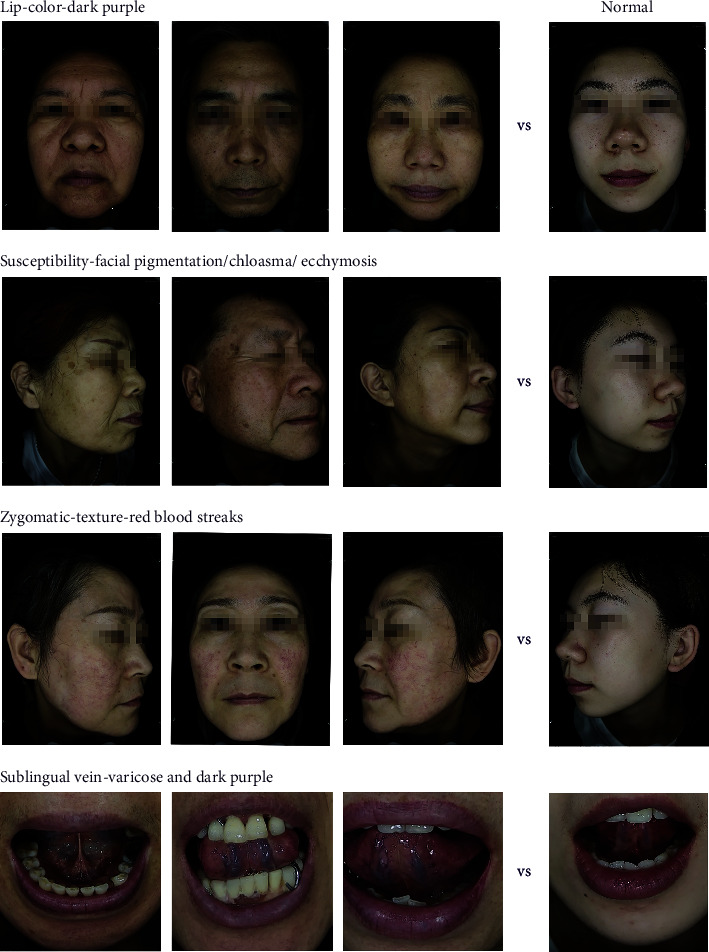
Images of typical facial and tongue features of BSC.

We display the representative pictures of facial and tongue features included in the nomogram to form a better perceptual understanding, as shown in Figure 5.

## 4. Discussion

To the best of our knowledge, this is the first study to systematically summarize the existing knowledge, clinical experience, and expert consensus on tongue and facial features of nine types of TCM constitution. Through the Delphi method, we obtained an expert consensus in terms of a table consisting of 34 items for 8 types of constitution. This table provides important prior knowledge and expert experience for future related constitution identification research. In the process of expert consultation, we also found that not all constitutions have typical facial or tongue features. Thus, constitution identification from the objective dimension of only facial and tongue features is not comprehensive, and the contributions of facial and tongue features in the identification of BSC, PDC, and DHC are relatively large, while those in the identification of ISC, BC, and QDC are relatively low. Therefore, it is not sufficient to rely solely on facial and tongue features to identify all types of constitution. This idea is consistent with previous research that has shown that only objective physical indicators without subjective feeling information can reduce the accuracy of the machine to imitate the doctor's diagnosis of TCM constitution [32]. Nevertheless, we found that tongue and facial features are critical links in constitution identification and still have important diagnostic value for BSC and other sensitive constitution types, which are worthy of in-depth study.

Therefore, based on the items obtained from expert consensus, we developed and validated a diagnostic nomogram for identifying BSC. BSC represents a tendency toward stagnation and is positively associated with the severity of atherosclerosis [33]. Indeed, a high distribution for BSC in coronary atherosclerotic heart disease, endometriosis, and stroke has been found in a literature review of 1639 clinical studies [2]. Early detection of BSC is of great significance to the prevention of related diseases. Based on the result of expert consensus, the developed nomogram incorporates six items: sex, hair volume, lip-color-dark purple, susceptibility-facial pigmentation/chloasma/ecchymosis, zygomatic-texture-red blood streaks, and sublingual vein-varicose and dark purple. Incorporating the objective facial, hair, and tongue features into an easy-to-use nomogram facilitates the individualized diagnosis of BSC. The shared characteristic of these features is that they are all objective indicators and do not need to be based on the patient's own feelings, which effectively reduces the bias of CCMQ results due to the difference in patient's subjective understanding [11]. The good performance of the nomogram indicated the feasibility of constitution identification through objective features.

Another point worth paying attention to is the quantification of feature indicators. In the past, traditional Chinese medicine diagnostics were often judged by general pathological characteristics; however, in actual clinical situations, it is rare for patients to have all of the typical pathological characteristics simultaneously. Therefore, it is necessary to further model and quantitatively evaluate the pathological signs that appear. In this study, we adopted a visual analog scale method of 0–10 points to quantify the degree of feature and develop a nomogram to calculate the risk of BSC.

This study has some limitations. First, doctors' scoring of indicators is subjective, and opinions of doctors may vary. To address this problem, we set up three experienced TCM doctors to simultaneously score the indicators of the subject and took the middle score as the result. Moreover, the images of the face and tongue were taken at the same time for better review and comparison in case of strong disagreement in the scoring. Second, the sample size is relatively small. To avoid the inconsistency of image standards caused by different image collection machines, all current images were collected by a single machine. Although this guarantees the comparability of images, it sacrifices the possibility of collecting a large number of samples in a short time. To deal with this problem, we will continue to increase the sample size in the future to provide adequate data preparation for machine learning training.

Inspection is an important part of the diagnosis of TCM constitution. Through our research, we have summarized the tongue and facial features of TCM constitution, providing theoretical basis and clinical experience for objective identification. Via the Delphi survey, we also realized that TCM constitution has different sensitivity to different kinds of health information, and accurate constitution recognition is bound to be based on multidimensional diagnostic information, which should include tongue and face information. In the future, we will strengthen the research on tongue and face information recognition. Using large samples of high-quality tongue and face images, we will strive to achieve an effective image diagnostic method and increase the proportion of objective indicators in constitution identification, and some information on symptoms will be included for symptom-sensitive constitution types. The development of artificial intelligence technology provides opportunities for TCM diagnosis. Under the guidance of TCM theory, better collection of targeted diagnostic information may provide greater benefits for the research on intelligent identification of TCM constitution.

## Figures and Tables

**Table 1:** Judgment basis of expert consultation based on the Delphi method.

Basis for judgment	Degree of influence on expert judgment		
	Large	Medium	Small
Theoretical analysis	0.3	0.2	0.1
Practical experience	0.5	0.4	0.3
Domestic and foreign references	0.1	0.1	0.1
Subjective intuition	0.1	0.1	0.1

**Table 2:** General information of experts (*n* = 11).

Item		*n*	%
Education	Master's degree	1	9
	Doctorate degree	10	91

Title	Chief physician/professor/researcher	5	45
	Associate chief physician/associate professor/associate researcher	3	27
	Attending physician/lecturer/assistant researcher	3	27

Major	TCM body constitution	8	73
	TCM prevention and treatment of disease	2	18
	TCM diagnostics	1	9

Experience in field (years)	5–10	3	27
	10–15	3	27
	20–25	2	18
	>30	3	27

Region	Beijing	9	82
	Shanghai	1	9
	Shenzhen	1	9

**Table 3:** Evaluation of the importance of facial features in identifying body constitution (*n* = 11).

Item	Item	Importance value (*m* ± SD)			Coefficient of variation (SD/*m*)
Overview	TCM body constitution	7.73	±	1.01	0.13
BC	Balanced constitution	6.82	±	1.83	0.27
QDC	Qi-deficiency constitution	7.64	±	1.50	0.20
YADC	Yang-deficiency constitution	7.09	±	1.64	0.23
YIDC	Yin-deficiency constitution	7.82	±	1.89	0.24
PDC	Phlegm-dampness constitution	8.82	±	1.47	0.17
DHC	Damp-heat constitution	8.82	±	1.40	0.16
BSC	Blood-stasis constitution	8.91	±	1.22	0.14
QSC	Qi-stagnation constitution	7.46	±	2.16	0.29
ISC	Inherited specific constitution	4.64	±	1.86	0.40

**Table 4:** Result of the degree of concentration of expert opinions.

TCM constitution type	Item	Round 1			Round 2			
		Importance value (*m* ± SD)	Coefficient of variation (SD/m)	Percentage of importance ≥4	Importance value (*m* ± SD)	Coefficient of variation (SD/m)	Percentage of importance ≥4	Coefficient of authority (*m*)
BC	Face-color-ruddy	4.27 ± 0.65	0.15	0.91	4.20 ± 0.63	0.15	0.9	0.94
	Eye-gaze-vigorous	4.65 ± 0.50	0.11	1.00	4.50 ± 0.53	0.12	1.0	0.92
	Nose-color-bright	4.18 ± 0.75	0.18	0.82	3.80 ± 0.63 (—)	0.17	0.7	0.79
	Lip-color-ruddy	4.27 ± 0.90	0.21	0.91	4.00 ± 0.67	0.17	0.8	0.87
	Tongue-color-light red	4.18 ± 0.60	0.14	0.91	4.20 ± 0.79	0.19	0.8	0.91

QDC	Face-color-yellow	4.00 ± 0.77	0.19	0.73	3.80 ± 1.03 (—)	0.27	0.6	0.92
	Face-color-pale	3.81 ± 0.75	0.20	0.82	3.80 ± 0.92 (—)	0.24	0.7	0.91

YADC	Face-color-pale	4.36 ± 0.50	0.12	1.00	4.20 ± 0.63	0.15	0.9	0.87
	Lip-shape-fat	4.09 ± 0.70	0.17	0.82	4.60 ± 0.70	0.15	0.9	0.93

YIDC	Face-color-flush	4.36 ± 0.50	0.12	1.00	4.10 ± 0.74	0.18	0.8	0.92
	Zygomatic-color-flush	4.09 ± 0.70	0.17	0.82	4.00 ± 0.67	0.17	0.8	0.90
	Lip-color-red	4.09 ± 0.70	0.17	0.82	4.30 ± 0.67	0.16	0.9	0.92
	Tongue-color-red	3.82 ± 1.08	0.28	0.82	4.50 ± 0.71	0.16	0.9	0.96

PDC	Face-shape-fat	4.36 ± 0.67	0.15	0.91	4.00 ± 0.82	0.20	0.7	0.92
	Face-oily	4.36 ± 0.67	0.15	0.91	4.20 ± 0.79	0.19	0.8	0.93
	Forehead-oily	4.18 ± 0.87	0.21	0.73	4.00 ± 0.67	0.17	0.8	0.90
	Eyelid-shape-swelling	4.36 ± 0.50	0.12	1.00	4.40 ± 0.52	0.12	1.0	0.90
	Tongue-coating-texture-thick	3.91 ± 1.14	0.29	0.82	4.30 ± 0.82	0.19	0.8	0.91

DHC	Face-dirty and stasis	4.00 ± 1.10	0.27	0.82	4.40 ± 0.70	0.16	0.9	0.93
	Face-oily	4.55 ± 0.69	0.15	0.91	4.60 ± 0.70	0.15	0.9	0.94
	Nose-oily	4.18 ± 0.87	0.21	0.73	4.00 ± 0.67	0.17	0.8	0.89
	Susceptibility-brandy nose	4.18 ± 0.98	0.23	0.82	3.70 ± 0.67 (—)	0.18	0.6	0.85
	Susceptibility-facial acne	4.46 ± 0.69	0.15	0.91	4.50 ± 0.53	0.12	1.0	0.97
	Tongue-coating-color-yellow	4.00 ± 0.45	0.11	0.91	4.70 ± 0.48	0.10	1.0	0.97
	Tongue-coating-texture-thick	4.18 ± 0.60	0.14	0.91	4.00 ± 0.82	0.20	0.9	0.95

BSC	Face-color-dark	4.55 ± 0.52	0.11	1.00	4.50 ± 0.71	0.16	0.9	0.91
	Face-color-black	4.18 ± 0.75	0.18	0.82	4.20 ± 0.79	0.19	0.8	0.87
	Susceptibility-facial pigmentation	4.09 ± 0.94	0.23	0.82	4.30 ± 0.67	0.16	0.9	0.94
	Susceptibility-ecchymosis	4.36 ± 0.50	0.12	1.00	4.50 ± 0.53	0.12	1.0	0.89
	Susceptibility-chloasma	4.18 ± 0.60	0.14	0.91	4.00 ± 0.82	0.20	0.7	0.88
	Zygomatic-texture-red blood streaks	4.09 ± 0.70	0.17	0.82	4.00 ± 0.82	0.20	0.7	0.84
	Cheek-texture-red blood streaks	4.00 ± 0.77	0.19	0.73	3.60 ± 0.52 (—)	0.14	0.6	0.77
	Eyelid-dark circles	4.09 ± 0.54	0.13	0.91	4.30 ± 0.48	0.11	1.0	0.94
	Lip-color-dark purple	4.36 ± 0.50	0.12	1.00	4.30 ± 0.48	0.11	1.0	0.92
	Tongue-color-dark purple	4.46 ± 0.52	0.12	1.00	4.60 ± 0.52	0.11	1.0	0.94
	Tongue-texture-bruise	4.36 ± 0.67	0.15	0.91	4.40 ± 0.52	0.12	1.0	0.94
	Sublingual-vein-varicose and dark purple	4.55 ± 0.52	0.11	1.00	4.70 ± 0.48	0.10	1.0	0.95
	Tongue edge-color-blue purple	4.00 ± 1.00	0.25	0.73	3.80 ± 0.79 (—)	0.21	0.6	0.84

SQC	Depressed appearance	4.55 ± 0.69	0.15	0.91	4.80 ± 0.42	0.09	1.0	0.95
	Saliva lines on both sides of the tongue	—	—	—	3.50 ± 0.71 (—)	0.20	0.4	0.80

ISC	Susceptibility-dermatitis manifestations	—	—	—	4.20 ± 0.79	0.19	0.8	0.90

Note: (—) means that the item was eliminated in the second round.

**Table 5:** Characteristics of participants in the primary and validation groups.

Characteristic	Primary group			Validation group		
	BSC (−)	BSC (+)	*P*	BSC (−)	BSC (+)	*P*
	*n* = 152	*n* = 62		*n* = 63	*n* = 19	
Sex			<0.01^*∗*^			0.03^*∗*^
Male	53 (34.9%)	6 (9.7%)		19 (30%)	1 (5%)	
Female	99 (65.1%)	56 (90.3%)		44 (70%)	18 (95%)	
Age (mean ± SD)	42.6 ± 11.5	50.0 ± 11.9	<0.01^*∗*^	41.9 ± 12.9	48.6 ± 11.1	0.05
BMI			0.13			0.48
BMI < 18.5	13 (8.6%)	4 (6.5%)		6 (10%)	1 (5%)	
18.5 < BMI ≤ 24	108 (71.1%)	37 (59.7%)		40 (63%)	10 (53%)	
24 < BMI ≤ 28	30 (19.7%)	19 (30.6%)		16 (25%)	7 (37%)	
BMI > 28	1 (0.7%)	2 (3.2%)		1 (2%)	1 (5%)	
Hair volume			<0.01^*∗*^			0.19
Less	9 (5.9%)	21 (33.9%)		4 (6%)	4 (21%)	
Normal	113 (74.3%)	34 (54.8%)		47 (75%)	12 (63%)	
More	30 (19.7%)	7 (11.3%)		12 (19%)	3 (16%)	
Hair oil secretion			<0.01^*∗*^			0.09
Dry	14 (9.2%)	19 (30.6%)		5 (8%)	4 (21%)	
Normal	119 (78.3%)	26 (41.9%)		49 (78%)	10 (53%)	
Oily	19 (12.5%)	17 (27.4%)		9 (14%)	5 (26%)	
Face-color-black	0.8 ± 1.4	1.2 ± 1.5	0.04^*∗*^	0.7 ± 1.6	0.9 ± 1.4	0.69
Face-color-dark	1.3 ± 1.7	2.2 ± 1.9	<0.01^*∗*^	1.2 ± 1.8	2.2 ± 1.5	0.04^*∗*^
Lip-color- dark purple	1.5 ± 2.0	2.8 ± 2.1	<0.01^*∗*^	1.3 ± 1.9	2.1 ± 2.0	0.12
Susceptibility-facial pigmentation/chloasma/ecchymosis	2.1 ± 2.0	3.7 ± 1.9	<0.01^*∗*^	2.5 ± 2.1	4.7 ± 2.1	<0.01^*∗*^
Eyelid-dark circles	1.7 ± 1.3	2.2 ± 1.5	<0.01^*∗*^	1.9 ± 1.1	2.4 ± 1.6	0.15
Zygomatic-texture-red blood streaks	0.2 ± 0.5	0.6 ± 1.2	<0.01^*∗*^	0.3 ± 0.7	0.5 ± 1.2	0.24
Tongue-color-dark purple	0.8 ± 1.5	1.9 ± 2.2	<0.01^*∗*^	0.8 ± 1.7	1.7 ± 1.6	0.06
Tongue-texture-bruise	0.2 ± 0.9	0.5 ± 1.5	0.03^*∗*^	0.2 ± 0.8	0.3 ± 0.7	0.61
Sublingual vein-varicose and dark purple	1.9 ± 1.5	3.8 ± 1.7	<0.01^*∗*^	2.0 ± 1.9	4.7 ± 1.8	<0.01^*∗*^

Note: ^*∗*^*P* value <0.05.

## Data Availability

All cleaned data we used in the article are transparent and available upon request by contact with the corresponding authors.
